# Effective Tobacco Control in Washington State: A Smart Investment for Healthy Futures

**Published:** 2007-06-15

**Authors:** Julia A. Dilley, Kristen Rohde, Clyde Dent, Michael J Stark, Michael J Boysun, Terry Reid

**Affiliations:** Program Design and Evaluation Services, Multnomah County Health Department/Oregon Department of Human Services; Program Design and Evaluation Services, Multnomah County Health Department, Oregon Department of Human Services, Portland, Ore; Program Design and Evaluation Services, Multnomah County Health Department, Oregon Department of Human Services, Portland, Ore; Program Design and Evaluation Services, Multnomah County Health Department, Oregon Department of Human Services, Portland, Ore; Tobacco Prevention and Control Program, Washington State Department of Health, Olympia, Wash; Tobacco Prevention and Control Program, Washington State Department of Health, Olympia, Wash

## Abstract

**Background:**

Tobacco use remains the leading cause of preventable death in the United States. Following the 1998 Master Settlement Agreement with the tobacco industry, Washington State dedicated substantial funding to the creation of a statewide, comprehensive tobacco control program. This report documents the history and observed effectiveness of that program.

**Context:**

In 2000, the Washington legislature allocated $100 million out of the first Master Settlement payment of $320 million to tobacco control. The comprehensive tobacco control program was launched late that same year with an annual budget of $15 million.

**Methods:**

We used existing data from state and national health behavior surveillance systems to describe smoking prevalence among adults and youth. For adult measures, we used data from the Washington State Behavioral Risk Factor Surveillance System and the National Health Interview Survey. For youth measures, we used data from the Washington State Healthy Youth Survey and the national Monitoring the Future survey. We used the National Cancer Institute's "Joinpoint" software to compare trends.

**Consequences:**

Between 1990 and 2001, adult smoking prevalence in Washington was nearly unchanged, as it was in the United States as a whole. However, from 2001, one year after Washington instituted its comprehensive tobacco control program, to 2005, the prevalence of smoking among adults in Washington declined significantly from 22.5% to 17.6%, and by a significantly larger amount than it did nationally during the same period (22.7% to 20.9%). In addition, the prevalence of youth smoking also declined faster in Washington than it did nationally; for example, from 2000 to 2004, smoking prevalence among 8th graders declined from 12.5% in 2000 to 7.8% in 2004 in Washington but only from 12.2% in to 9.3% nationally.

**Interpretation:**

Significant reductions in smoking prevalence among Washington residents following the implementation of a comprehensive tobacco control program funded at a level near that recommended by the Centers for Disease Control and Prevention indicate that tobacco control programs are an effective investment for states committed to improving public health.

## Background

Tobacco use and exposure to secondhand smoke are widely considered to be leading causes of preventable disease and death in the United States (1). To help reduce the prevalence of tobacco use and exposure to secondhand smoke, the Centers for Disease Control and Prevention (CDC) described a "best practices" framework, including recommended funding levels, for comprehensive state tobacco control programs (2). This framework was modeled on successful programs to reduce smoking that were implemented in California and Massachusetts, as well as on research findings. Individual components of the framework are designed to work synergistically with changes in government policies aimed at reducing tobacco use, such as increasing taxes on tobacco products and prohibiting smoking in specified public areas.

Following the 1998 Master Settlement Agreement between states' attorneys general and the tobacco industry, Washington State dedicated substantial funding to tobacco control and used the CDC framework to design a comprehensive state program. Five years after the program was implemented in 2002, policymakers are considering what level of funding to allocate for ongoing support of the program.

Recent research has demonstrated that there is a dose-response relationship between investment in tobacco control and the extent to which tobacco usage rates decline. For example, the results of a study that correlated state trends in cigarette consumption with state per capita expenditures on major tobacco control programs showed that increased investment in tobacco control was associated with reductions in smoking and concluded that "larger, more established programs may have a larger dollar for dollar impact" (3). Similarly, an analysis of national youth data using a variety of statistical models also found that per capita tobacco control expenditures were inversely associated with smoking prevalence and daily cigarette consumption among youths and estimated that the prevalence of youth smoking would be 3% to 14% lower today if all states had spent the minimum amount recommended by CDC (4).

In this report, we describe the investment in and implementation history of tobacco control programs in Washington State, assess the effectiveness of those programs over time, and identify critical populations remaining in need of support.

## Context

**Figure 1:**
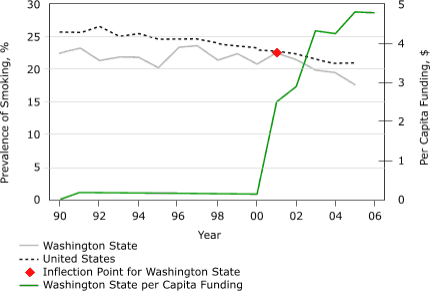
Smoking prevalence rates among adults in Washington State and the United States and tobacco control funding per capita in Washington State, 1990 through 2005. Data for Washington State are from the Washington State Behavioral Risk Factor Surveillance System; data for the United States are from the National Health Interview Survey. The point of inflection is the point at which the slope of a line changes significantly.

Table 1 summarizes the history of tobacco control programs, cigarette taxation, and major tobacco-related legislation in Washington; Figure 1 shows the total per capita funding for statewide tobacco control programs from 1990 through 2005. In 1990, before implementation of any substantial tobacco control activities in Washington, 22.5% of Washington adults smoked.

**Table:** 

**Year**	**Adult Smoking Prevalence, Washington State, %**	**Adult Smoking Prevalence, United States, %**	**Per Capita Funding for Tobacco Control Programs, Washington State, $**
1990	22.5	25.5	0.04
1991	23.3	25.7	0.20
1992	21.4	26.5	0.19
1993	21.9	25.0	0.19
1994	21.9	25.5	0.19
1995	20.2	24.7	0.18
1996	23.4	24.7	0.18
1997	23.8	24.7	0.18
1998	21.4	24.1	0.17
1999	22.4	23.5	0.17
2000	20.7	23.2	0.19
2001	22.5	22.7	2.51
2002	21.5	22.4	2.90
2003	19.8	21.6	4.30
2004	19.5	20.9	4.25
2005	17.6	20.9	4.81

In 1991, Washington was one of 17 states to receive funding from Project ASSIST (American Stop Smoking Intervention Study), a state-based tobacco control study implemented by the National Cancer Institute (5). The goal of ASSIST was to change the social, cultural, economic, and environmental factors that influence smoking behavior by policy changes in three areas: increasing tobacco taxes, limiting youth access to tobacco, and promoting smoke-free environments. ASSIST funds were used to establish a statewide coalition called Tobacco Free Washington and to implement tobacco control activities in several large counties. In 1993, Washington passed a state law that required retailers to be licensed to sell tobacco products, and a portion of the license fee was dedicated to tobacco control in the communities where the retailers were located. From 1991 through 1999, Washington also passed laws to restrict smoking in workplaces and modestly increased taxes on tobacco products.

In 1998, the Master Settlement Agreement was negotiated between the tobacco industry and states' attorneys general, led by then Washington Attorney General Christine Gregoire. Washington State received its first $320 million settlement payment in 2000, and the state legislature allocated $100 million of that payment to support a comprehensive state tobacco control program. The state then established the Washington Tobacco Prevention and Control Council to develop a comprehensive tobacco control plan for how allocated funds were to be used (6). The plan was grounded in the CDC framework and developed on the basis of advice from hundreds of stakeholders in topical working groups. Washington State began to implement the plan in the fall of 2000.

Table 2 summarizes the CDC framework for tobacco control, including the CDC-recommended percentage of tobacco control funds to be spent on individual program components and the percentage of Washington State's current funding spent in each component area. Currently, the overall program is funded at $26.3 million per year, which is approximately 70% of the CDC-recommended minimum level of $37.4 million (calculated by multiplying the 1999 minimum per capita recommendation by the 2005 Washington State population [7]).

Washington State spends approximately the CDC-recommended proportion of its tobacco control budget on school programs, statewide programs, surveillance/evaluation, and administration (Washington State, 34%; CDC recommendation, 29%). Washington's school programs focus on implementing CDC's best practices for school-based tobacco prevention (8) in grades 5 through 9. To reach targeted population groups statewide, leadership groups are funded to coordinate tobacco control activities in minority communities and American Indian tribal areas, as well as activities by businesses and the University of Washington Public Health Training Program; funds have also been used to support a long-established statewide multidisciplinary substance abuse/violence prevention training/networking conference and to support surveillance and evaluation activities, including targeted evaluation activities and the enhancement of existing public health surveillance systems such as the Behavioral Risk Factor Surveillance System (BRFSS) and the Healthy Youth Survey (HYS).

Washington State spends more than the CDC-recommended portion of its tobacco control budget on community-based programs (Washington State, 22%; CDC recommendation, 14%) and counter-marketing (Washington State, 31%; CDC recommendation, 18%). The state funds community programs in each of Washington's 39 counties and in 27 of 29 federally recognized American Indian tribal nations within the state; it also provides support for regional training sessions and meetings that support other program components. The state has sponsored three paid media campaigns to discourage tobacco use (tobacco use prevention, smoking cessation/quit line promotion, and secondhand smoke awareness); it has also engaged in tobacco-related media outreach, promoted or sponsored events to discourage tobacco use, and served as a clearinghouse for public education and social marketing materials.

Washington spends less than the CDC-recommended proportion of its available state tobacco control budget on disease-related programs, the enforcement of smoking prohibitions, and smoking cessation programs (Washington State, 13%; CDC recommendation, 39%). When the Washington Tobacco Prevention and Control Council developed the state plan for tobacco control, Washington already had an advanced cancer registry; some tobacco control funds have been applied to support the "planned care model" approach to changing health care systems, which includes integrating clinical best practices for tobacco use cessation to treat people with chronic disease. Funding for the enforcement of youth access laws has been shared with the state Liquor Control Board, which has authority to inspect tobacco retailers, but most of the enforcement authority for statewide youth access and clean indoor air policies is at the local level; the higher rate of funding for community programs reflects support for this enforcement. The cessation component of the state tobacco control program includes a statewide toll-free quit line, which provides support to all smokers who want help in quitting as well as intensive counseling and nicotine replacement therapy to low-income and uninsured tobacco users; statewide training sessions to mobilize community programs to encourage health care systems to address tobacco use and to adopt clinical best practices for assisting patients with smoking cessation; and targeted funding to support smoking cessation within substance-abuse treatment programs.

## Methods

We used state and national surveillance data to evaluate changes in tobacco use prevalence among adults and youth from 1990 through 2005 and to determine whether the implementation of Washington State's comprehensive tobacco control program was associated with a significant reduction in smoking prevalence.

To describe trends in smoking prevalence among adults, we used data from the Washington State BRFSS (www.cdc.gov/brfss) and the National Health Interview Survey (NHIS) (www.cdc.gov/nchs/nhis.htm). We then compared adult smoking prevalence trends for Washington with those for the nation from 1990 through 2005 using the National Cancer Institute's Joinpoint software (9), which calculates slope values (indicating the absolute change in prevalence per year) and also identifies "points of inflection" where the slope of a trend line changes significantly. To describe and compare prevalence trends among youth, we used data on Washington State youth from the Washington State HYS (www3.doh.wa.gov/hys), an established youth risk behavior survey based largely on the national Youth Risk Behavior Survey (www.cdc.gov/yrbs) (10) and the Communities that Care survey (11), and we used data on all U.S. youth from the Monitoring the Future (MTF) survey (www.monitoringthefuture.org/). The HYS is administered to 6th, 8th, 10th, and 12th graders during the fall of even years. The MTF is administered to 8th, 10th, and 12th graders during the spring of every year. In both surveys, "current smoking" is defined as having smoked any cigarettes at any time during the previous 30 days. We compared smoking trends among Washington youth with those among U.S. youth for 2000 through 2005 only, because Washington's youth survey methodology was not well established before 2000.

## Consequences

Figure 1 illustrates the trends in adult smoking prevalence in Washington and in the nation. No point of inflection was identified in the national trend. From 1990 to 2005, the national prevalence of smoking among adults declined significantly from 25.5% to 20.9%, an average of 0.33 percentage points per year (slope of trend line, −0.35; 95% confidence interval [CI], −0.29 to −0.41). For Washington State, however, 2001 was a point of inflection in the prevalence of adult smoking. From 1990 to 2001, the prevalence of smoking in Washington State was stable (22.5% for both 1990 and 2001), but from 2001 to 2005, it declined by an average of 1.22 percentage points per year (slope of trend line, −1.05; 95% CI, −0.45 to −1.64) to 17.6% in 2005, which is both a significant decline and significantly greater than the decline observed nationally during the same period. Thus the smoking prevalence among Washington State adults began to decline by significantly more than that among U.S. adults beginning just 1 year after the implementation of a well-funded comprehensive statewide program.

**Figure 2:**
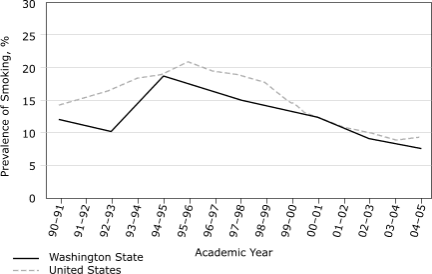
Smoking prevalence rates among 8th-grade youths in Washington State and in the United States, 1990 through 2005. Data for Washington State are from the Washington State Healthy Youth Survey; data for the United States are from the national Monitoring the Future survey.

Figure 2 illustrates the trends in smoking prevalence among 8th graders in Washington State and nationally. The national rate declined by an average of 0.72 percentage points per year from spring 2001 to spring 2005 (slope of trend line, −0.73; 95% CI, −0.44 to −1.02), while the prevalence in Washington declined by an average of 1.18 percentage points per year  (slope of trend line, −1.00; 95% CI, −0.38 to −1.63). The absolute decline of 4.7 percentage points in Washington State, from 12.5% in fall 2000 to 7.8% in fall 2004, exceeded the absolute national decline of 2.9 percentage points, from 12.2% in the spring of 2001 to 9.3% in the spring of 2005. During these periods, smoking prevalence also fell by 6.8 percentage points among 10th graders in Washington State, compared with 6.4 percentage points among all 10th graders in the United States, and by 7.9 percentage points among 12th graders in Washington State, compared with 6.3 percentage points among all 12th graders in the United States. Although these differences were not statistically significant, they do suggest that the rate of decline in the prevalence of youth smoking was faster in Washington than in the nation as a whole for the period after Washington implemented its comprehensive tobacco control program. A separate analysis of cohort growth rates in smoking prevalence among Washington students between the 6th and 12th grades from 1991 through 2005 (data not shown) also indicated that the cohort growth rate in Washington was lower than that in the nation.

**Table:** 

**Academic Year**	**Smoking Prevalence, 8th-Grade Youths, Washington State, %**	**Smoking Prevalence, 8th-Grade Youths, United States, %**
1990-91	12.1	14.3
1991-92	Not Available	15.5
1992-93	10.3	16.7
1993-94	Not Available	18.6
1994-95	18.8	19.1
1995-96	Not Available	21.0
1996-97	Not Available	19.4
1997-98	15.2	19.1
1998-99	Not Available	17.5
1999-2000	Not Available	14.6
2000-01	12.5	12.2
2001-02	Not Available	10.7
2002-03	9.2	10.2
2003-04	Not Available	9.2
2004-05	7.8	9.3

Although Washington made some progress in instituting tobacco control policies and increasing tobacco taxes from 1990 through 2000, the prevalence of smoking did not decline significantly until after the state's substantial investment in a comprehensive tobacco control program. The prevalence of smoking among Washington adults declined from 22.4% in 1999, just before the launch of the program, to 17.6% in 2005. This means that there were approximately 205,000 fewer smokers in the state in 2005 than there would have been had smoking rates remained as they were before the comprehensive program was instituted. Researchers have estimated that this reduction in the number of smokers will result in future direct medical savings of $1.95 billion (12).

Despite the success of state tobacco control programs, a growing body of evidence suggests that policy makers are quick to use tobacco control money for other purposes (13) and to reduce or eliminate the funding even of successful tobacco control programs, such as those implemented in Florida and Massachusetts (14,15). Unfortunately, there is no evidence that progress in reducing tobacco use and exposure in states with comprehensive programs is sustainable after the programs are defunded, especially since the tobacco industry continues to aggressively increase its marketing expenditures and activities (16). In fact, almost immediately following the defunding of a successful state tobacco control program in Minnesota, progress in reducing smoking among youth began to subside (17). Similarly, the results of a recent CDC study suggest that stalled progress in reducing the national prevalence of smoking among adults may be at least partially attributable to substantial reductions in state resources dedicated to tobacco control (18).

**Figure 3:**
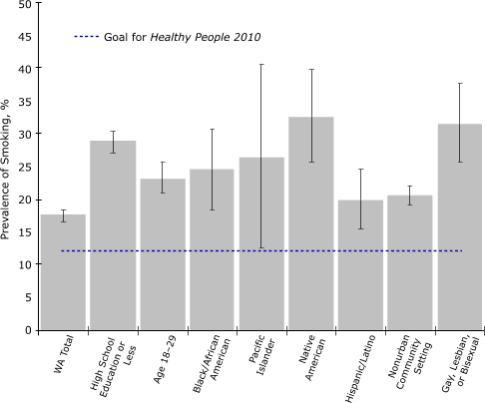
Smoking prevalence rates among demographic groups in Washington State that have prevalence rates higher than the state average. The category "Hispanic/Latino" includes English-speaking people of any race. Error bars represent 95% confidence intervals. Data are from the Washington State Behavioral Risk Factor Surveillance System, 2005.

**Table:** 

**Demographic Group**	**Adult Prevalence Rate, Washington State, %**
Washington State	17.6 (16.9-18.4)
High school education or less	28.9 (27.4-30.5)
Age 18-29	23.3 (21.2-25.6)
Black/African American	24.7 (19.4-30.9)
Pacific Islander	26.6 (16.2-40.6)
Native American	32.6 (26.2-39.6)
Hispanic/Latino	20.1 (16.3-24.7)
Nonurban community setting	20.5 (19.2-21.9)
Gay, lesbian, or bisexual	31.6 (26.1-37.6)

Using 2005 Washington BRFSS data to identify demographic disparities in smoking prevalence among Washington adults, we found levels to be higher than average among those with a high school education or less; those aged 18 to 29; those living in nonurban zip-code areas (according to the Rural Urban Commuting Area [RUCA] classification (19); those who were gay, lesbian, or bisexual; and those who were black, Native American, Pacific Islander, or English-speaking Hispanic (Figure 3). Although Asians overall had a lower prevalence than the state average, studies in Washington State found smoking prevalence among Korean and Vietnamese men to be approximately 30% (18); thus, they represent a priority population. These populations appear less responsive to mainstream tobacco control activities and may require increasingly individualized approaches to be reached effectively (20,21). These populations are also expected to be a focus of targeted tobacco industry marketing as the industry's access to youth is reduced and the population at large becomes less receptive to its marketing efforts (22,23). Substantial sustained funding will be required to reach these groups with effective counter-marketing messages. The Washington tobacco control program is implementing evaluation activities to assess the extent to which program activities proven to be successful in the general population can also be also be used successfully in demographic subgroups with relatively high smoking rates; for example, the program recently examined quit line success rates and satisfaction among poor, minority, rural, and young adults (Dent CW, et al: Unpublished study described in a poster presented at the 2005 National Conference on Tobacco or Health, Chicago, Ill, May 2005).

## Interpretation

From 2000, when Washington established a comprehensive tobacco control program and funded it at a level approaching the minimum recommended by CDC, through 2005, the prevalence of smoking in Washington declined significantly among both adults and youth (and significantly more than in the nation as a whole).

Our findings indicate that a comprehensive tobacco control program is an effective and cost-effective investment in public health and that a substantial sustained investment will produce better results than minimal investment. Additionally, the remaining population of Washington smokers may be more difficult to reach through mainstream public health channels, suggesting that funding for successful tobacco control programs must be sustained at substantial levels rather than decreased over time.

## Figures and Tables

**Table 1:** History of Tobacco Control Program Implementation, Tobacco Taxation, and Major Tobacco-Related Legislation in Washington State, 1990–2006

Year	Program Implementation, Taxes, Legislation
**1990: No dedicated tobacco control program**	Tax: Baseline state cigarette tax is $0.34/pack.Legislation: State prohibits use of tobacco on school property.
**1991 to 1999: ASSIST (American Stop Smoking Intervention Study) Program**	
1993	Tax: Cigarette tax increased $0.20 to $0.54/pack.Legislation: Tobacco retailer licensing requirement established; youth tobacco prevention account created for community grants; tobacco sample distribution banned except in adult-only environments.
1994	Tax: Cigarette tax increased $0.025 to $0.565/pack.Legislation: Clean Indoor Air Act banned smoking in most indoor work environments; exceptions include restaurants/bars.
1995	Tax: Cigarette tax increased $0.25 to $0.815/pack.
1996	Tax: Cigarette tax increased $0.01 to $0.825/pack.
1997	Legislation: All tobacco products banned from school property.
1999	Program: The Tobacco Prevention and Control Council issued a plan for a comprehensive tobacco control program.
2000	Program: Legislature funded the comprehensive program at $15 million per year. Program launched in the fall of 2000.
**2001 to present: Comprehensive Program**	
2001	Program: Legislature funded comprehensive program at $17.5 million per year.
2002	Tax: Cigarette tax increased $0.60 to $1.425/pack in a voter initiative.Program: Tax increase used to increase comprehensive program funding to $26.3 million per year.Legislation: Possession of tobacco by people younger than age 18 years banned.
2005	Tax: Cigarette tax increased $0.60 to $2.025/pack.Legislation: Clean Indoor Air Act implemented in all workplaces, including restaurants and bars, as the result of a voter initiative.
2006	Legislation: All distribution of tobacco samples banned. (In June 2006, the ban was partially overturned so that it does not apply to cigarettes.)

**Table 2:** Comprehensive Tobacco Control Program Design, Washington State, 2005–2007

Component	Description	**CDC**-**Recommended Funding Level^a^**	Washington State Funding Level^b^
Community/tribal programs	Programs that build and support community coalitions to promote policies that discourage tobacco use (e.g., clean air, minors' access, and cessation coverage programs).	14%	22%
Tobacco-related disease programs	Programs that support the prevention, detection, and treatment of tobacco-related diseases (e.g., a cancer registry and CVD, asthma, and oral health programs).	6%	<1%
School programs	Programs designed to prevent smoking among students through strategies such as banning tobacco products on school property and incorporating tobacco-free health material in the school curriculum.	10%	12%
Enforcement	Programs that enforce established policies to discourage smoking.	6%	1%
Statewide programs	Tobacco control programs that engage organizations with statewide access to diverse communities or specific subgroups of the state population.	6%	7%
Counter marketing	Campaigns that counter tobacco marketing with health messages about the dangers of tobacco use.	18%	31%
Cessation	Programs that support smoking cessation interventions by health care providers, deliver population-based treatment (e.g., quit lines), and eliminate cost barriers to cessation treatment by providing underserved groups with free or subsidized services.	27%	12%
Surveillance/evaluation	Data collection systems used to monitor people's tobacco-related behaviors, attitudes, and health outcomes. Reports based on collected data allow policy makers to gauge the effectiveness of tobacco control programs.	9%	8%
Administration	The management structure that coordinates components of tobacco control programs, implements contracts, and engages multiple state leaders and agencies in tobacco program activities.	4%	7%
Total^c^	Not applicable.	$37.4 to $100.1 million	$26.3 million
Per capita	Not applicable.	$5.94 to $15.93	$4.19

CDC indicates Centers for Disease Control and Prevention; CVD, cardiovascular disease.

a Amount represents the percentage of the total amount recommended for state tobacco control funding.

b Amount represents the percentage of the total amount budgeted for state tobacco control (July 2005 through June 2007 biennial state budget).

c The CDC-recommended total funding for Washington was calculated by multiplying the minimum and maximum per capita recommendations by the 2005 Washington State population.
